# Hemosiderotic diffuse normolipemic plane xanthoma presented with skin hyperpigmentation

**DOI:** 10.1016/j.jdcr.2024.06.039

**Published:** 2024-07-24

**Authors:** Wanwarang Permpornsakul, Ploysyne Rattanakaemakorn, Korn Triyangkulsri

**Affiliations:** Division of Dermatology, Department of Medicine, Faculty of Medicine, Ramathibodi Hospital, Mahidol University, Bangkok, Thailand

**Keywords:** foam cells, hemosiderin, monoclonal gammopathy, multiple myeloma, non-Langerhans cell histiocytosis, xanthosiderohistiocytosis

## Introduction

Diffuse normolipemic plane xanthoma (DNPX) is commonly presented as xanthoderma or symmetric yellowish plaques that favor the neck, flexural folds, upper portion of the trunk, and periorbital region.^1^ The diagnosis of DNPX is crucial because of its frequent association with monoclonal gammopathy and multiple myeloma.^2^ Skin hyperpigmentation was not a presentation commonly described in the literature for plane xanthoma. The closest clinical presentation to plane xanthoma with hyperpigmentation was described in a case report as hemosiderotic xanthelasma, in which the patient presented with a yellowish discoloration and grayish central areas on the eyelids. However, the entity was characterized as a variant of xanthosiderohistiocytosis (XSH) or xanthoma disseminatum (XD) because of the presence of Touton giant cells and hemosiderophage.^3^ Herein, we report a case of hemosiderotic DNPX presented with generalized xanthoderma and skin hyperpigmentation.

## Case report

**Fig 1:**
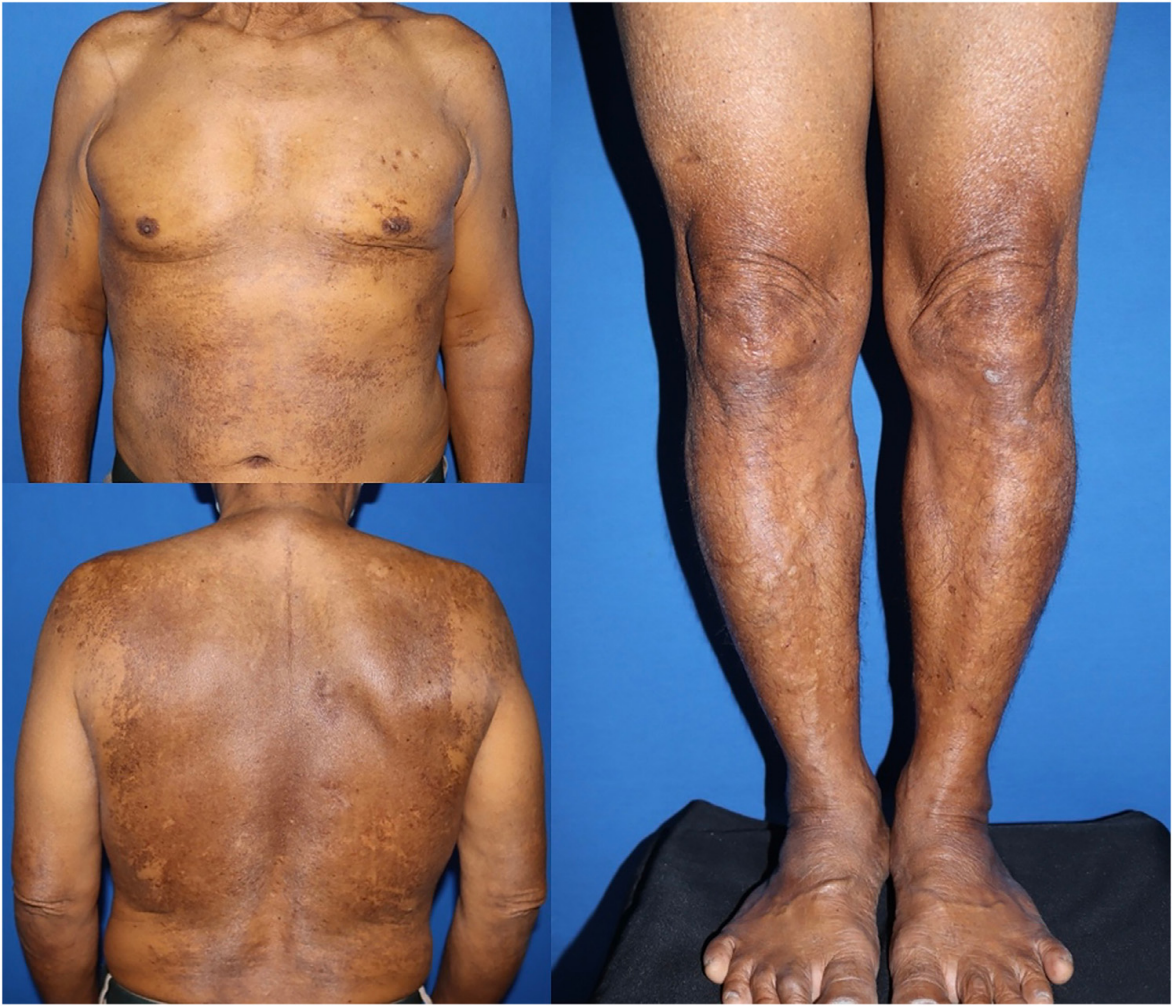
A 67-year-old man with generalized yellowish discoloration with overlying hyperpigmented macules and patches on the trunk and all extremities.

A 67-year-old man complained of progressive and generalized skin hyperpigmentation for 5 years without any other symptom. He denied any history of topical agent, herbal, sun, or heat exposure. Our patient had a medical condition of hypertension and benign prostatic hyperplasia, and he was receiving amlodipine, enalapril, doxazosin, and finasteride as part of his treatment. Dermatologic examination revealed generalized yellowish discoloration with overlying hyperpigmented macules and patches on the trunk and all extremities (Fig 1). There was no hepatosplenomegaly or lymphadenopathy.

**Fig 2:**
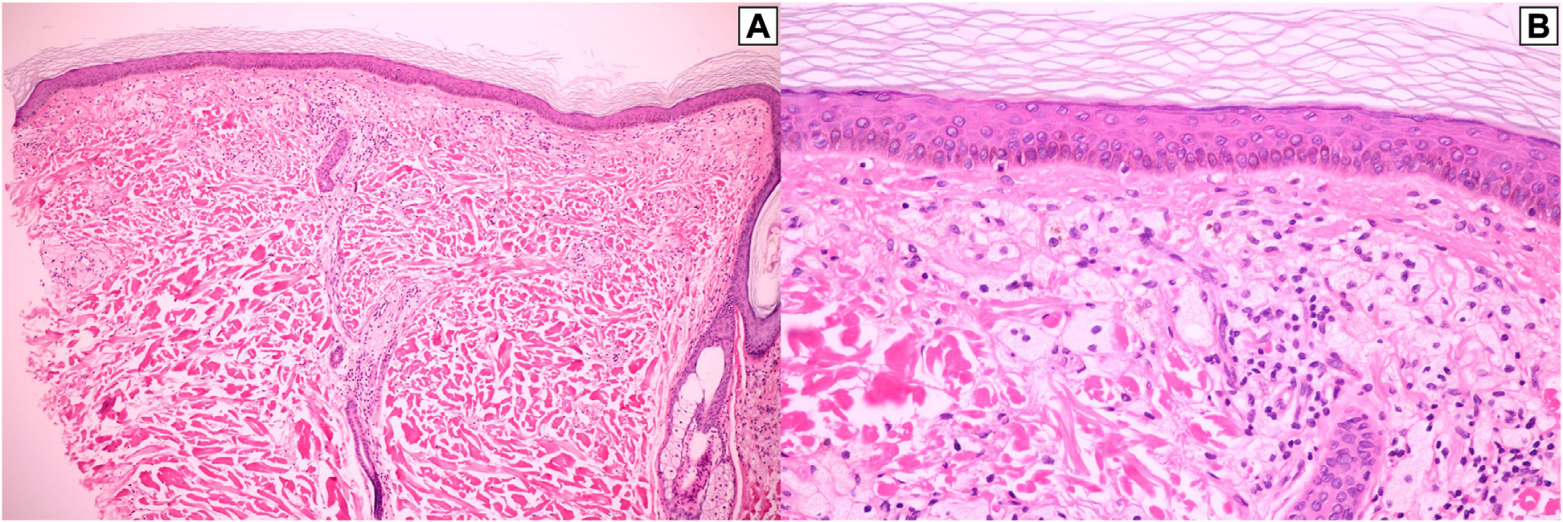
**A,** Band-like infiltration of cells in the papillary dermis. **B,** The cells composed of mainly foamy histiocytes admixed with sparse lymphocytic infiltration. No Touton giant cells or extracellular lipids were seen. (**A** and **B,** Hematoxylin-eosin stain; **A,** ×100; **B,** ×400.)

**Fig 3:**
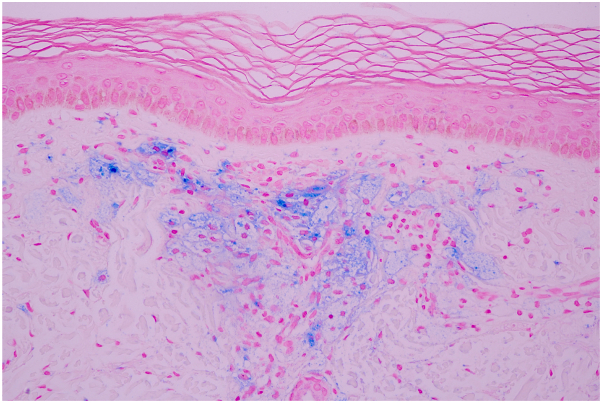
Perl’s stain highlighting hemosiderin in the cytoplasm of foamy histiocytes. (Original magnification: ×400.)

A skin biopsy taken from a lesion on his back demonstrated band-like infiltration of foamy histiocytes containing pigmented granules in the papillary dermis with sparse lymphocytic infiltration. No Touton giant cells or extracellular lipids were seen (Fig 2). Perl’s stain for hemosiderin was positive within the foam cells (Fig 3). A few extravasated erythrocytes were also found in the papillary dermis. The clinicopathologic findings were consistent with hemosiderotic DNPX. A complete blood count showed pancytopenia and high lactate dehydrogenase (266 U/L). His lipid profile, calcium, renal, liver, and thyroid functions were within the normal range. Serum protein electrophoresis demonstrated a monoclonal spike in the gamma region and was identified as an IgG κ on serum immunofixation electrophoresis. Serum free light chains revealed an increased κ level of 844.8 mg/L (normal, 3.3-19.4 mg/L) with an increased κ-to-λ ratio (844.8/13.4 = 62.9). On quantitative immunoglobulin analysis, IgG was elevated at 30.98 mg/mL (normal, 6.0-16.0 mg/mL). IgA and IgM were slightly decreased to 0.58 and 0.27 mg/mL, respectively (normal IgA, 0.7-4.0 mg/mL; normal IgM, 0.4-2.3 mg/mL). Bone marrow aspiration and biopsy revealed neoplastic plasma cells, which comprised approximately 30% of the marrow cellularity with κ-light chain restriction. Accordingly, the diagnosis of IgG κ multiple myeloma was confirmed.

**Fig 4:**
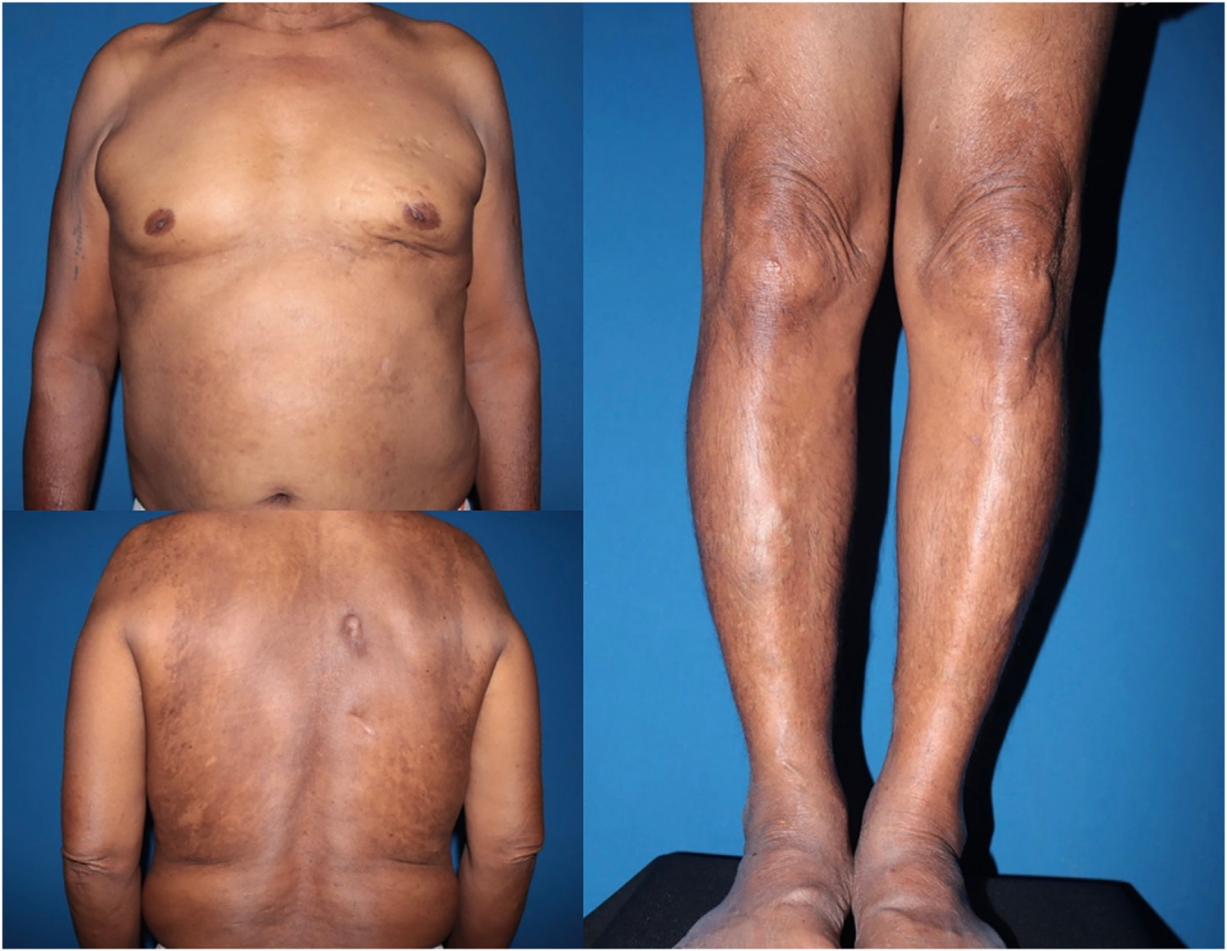
Clinical improvement in color and texture of the skin lesions after the treatment of multiple myeloma.

The patient then received bortezomib, cyclophosphamide, and dexamethasone therapy for 6 cycles for the multiple myeloma. After 10 months of treatment, his laboratory studies showed a good response. The complete blood count was restored to the normal range. No paraprotein was detected in the serum immunofixation electrophoresis. Serum free light chains revealed regular κ and λ levels of 16.83 and 16.34 mg/L, respectively, resulting in a normal κ-to-λ ratio. IgG, IgA, and immunoglobulin M also showed normal values of 8.97, 1.14, and 0.58 mg/mL, respectively. Correspondingly, there was a significant regression of the skin lesions with lessened xanthoderma and a lightening of the skin hyperpigmentation after the appropriate treatment of multiple myeloma (Fig 4).

## Discussion

DNPX is a rare subtype of non-Langerhans cell histiocytosis presented as symmetric yellowish plaques and known to be associated with hematologic disorders, particularly multiple myeloma and monoclonal gammopathy.^2^^,^^4^^,^^5^ Our case was a normolipemic man with IgG κ multiple myeloma who had diffuse yellowish discoloration overlying by hyperpigmented lesions, in which the histopathologic features suggested the diagnosis of DNPX but with unaccustomed hemosiderin presented in foamy histiocytes. Hyperpigmented lesions in xanthoma were previously described in XSH, an uncommon subtype of XD, with only a few cases reported to date.^3^^,^^6^, ^7^, ^8^, ^9^ One had a distinct clinical feature called hemosiderotic xanthelasma, which was identified as a localized variant of XSH of the eyelids.^3^ Systemic involvement, such as weight loss, photophobia, weakness, and neurologic, digestive, skeletal, and ocular findings, was also reported in those cases as in XD but not in our patient. Interestingly, 80% revealed the presence of IgG κ-type monoclonal gammopathy, as in our case. Apart from the difference in the clinical presentation from XSH and XD, our patient also showed no other histologic features of XSH or XD, such as the presence of Touton giant cells or dermal fibrosis. This makes the diagnosis of our case deviate toward hemosiderotic DNPX.

The pathogenesis of DNPX has not been completely illustrated. However, in gammopathy-associated plane xanthoma, monoclonal IgG is believed to bind to circulating low-density lipoprotein, making the antibody-low-density lipoprotein complex more susceptible to phagocytosis by macrophages.^5^ For XSH, it is hypothesized that there was a proliferation of histiocytes phagocytosing more iron than lipid.^7^ In the case of the recently aforementioned hemosiderotic xanthelasmas, the authors explained that the iron deposits in the cytoplasm of the cells could be the occurrence of microhemorrhages in the context of the patient taking an anticoagulant drug and the fact that the skin of the eyelids is thinner than in the rest of the body.^3^ This postulation may also be applicable in our case, as our patient has thrombocytopenia and a few extravasated erythrocytes were also found in the histology. However, further studies are needed to confirm this hypothesis.

There is no standard regimen for the treatment of DNPX, but several treatment options are currently available. Surgical excision, chemabrasion, dermabrasion, and ablative laser therapy such as the erbium:yttrium-aluminum-garnet laser were reported to be effective.^5^^,^^10^ However, these treatments also come with the risk of scarring. Our patient was treated with bortezomib, cyclophosphamide, and dexamethasone therapy for multiple myeloma for 6 cycles, with subsequent good clinical and laboratory responses. Concurrently, the patient’s hemosiderotic DNPX also showed a remarkable improvement in color and texture without any additional treatment.

## Conflicts of interest

None disclosed.
